# *xImpact*: Intelligent Wireless System for Cost-Effective Rapid Condition Assessment of Bridges under Impacts

**DOI:** 10.3390/s22155701

**Published:** 2022-07-29

**Authors:** Yuguang Fu, Yaoyu Zhu, Tu Hoang, Kirill Mechitov, Billie F. Spencer

**Affiliations:** 1 School of Civil and Environmental Engineering, Nanyang Technological University, Singapore 639798, Singapore; yuguang.fu@ntu.edu.sg; 2 CCCC Highway Bridges National Engineering Research Centre Co., Ltd., Beijing 100088, China; 3 Department of Civil Engineering, Tsinghua University, Beijing 100191, China; hoanganhtu.ams.hau@gmail.com; 4 Palo Alto Research Center, Palo Alto, CA 94304, USA; 5 Embedor Technologies, Champaign, IL 61820, USA; kmechitov@embedortech.com; 6 Department of Civil and Environmental Engineering, University of Illinois at Urbana-Champaign, Urbana, IL 61820, USA; bfs@illinois.edu

**Keywords:** bridge impact detection, rapid condition assessment, wireless smart sensors, structural health monitoring, artificial neural network

## Abstract

Bridge strikes by over-height vehicles or ships are critical sudden events. Due to their unpredictable nature, many events go unnoticed or unreported, but they can induce structural failures or hidden damage that accelerates the bridge’s long-term degradation. Therefore, always-on monitoring is essential for deployed systems to enhance bridge safety through the reliable detection of such events and the rapid assessment of bridge conditions. Traditional bridge monitoring systems using wired sensors are too expensive for widespread implementation, mainly due to their significant installation cost. In this paper, an intelligent wireless monitoring system is developed as a cost-effective solution. It employs ultralow-power, event-triggered wireless sensor prototypes, which enables on-demand, high-fidelity sensing without missing unpredictable impact events. Furthermore, the proposed system adopts a smart artificial intelligence (AI)-based framework for rapid bridge assessment by utilizing artificial neural networks. Specifically, it can identify the impact location and estimate the peak force and impulse of impacts. The obtained impact information is used to provide early estimation of bridge conditions, allowing the bridge engineers to prioritize resource allocation for the timely inspection of the more severe impacts. The performance of the proposed monitoring system is demonstrated through a full-scale field test. The test results show that the developed system can capture the onset of bridge impacts, provide high-quality synchronized data, and offer a rapid damage assessment of bridges under impact events, achieving the error of around 2 m in impact localization, 1 kN for peak force estimation, and 0.01 kN·s for impulse estimation. Long-term deployment is planned in the future to demonstrate its reliability for real-life impact events.

## 1. Introduction

**Figure 1:**
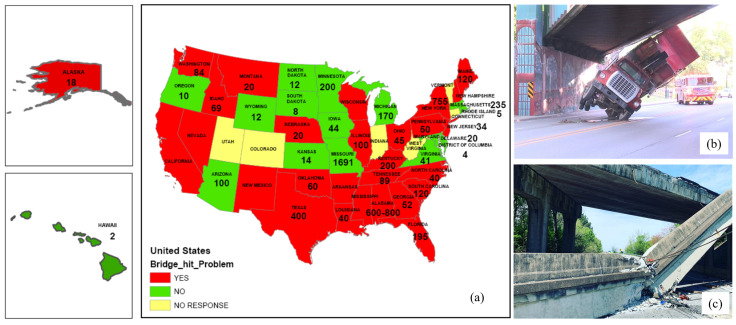
Bridge impact problem across the U.S.: (**a**) a nationwide survey by CUNY researchers [2], (**b**) railroad bridge impact at South Bend, IN, 2018, (**c**) a highway bridge impact in Chattanooga, TN, 2019.

Bridge strikes by over-height vehicles or ships are identified as critical sudden events. Around 5000 over-height vehicle bridge hits annually occur in the U.S., resulting in over $100 million worth of damage to public and personal property [1]. Figure 1 shows the number of bridge hits annually in each state of the U.S. and the severity assessment from each State DOT (Department of Transportation). Over 30 states in the U.S. consider bridge strikes to be a major problem [2]. One typical railroad bridge impact and one typical highway bridge impact are shown in Figure 1b,c. Similarly, the railroad infrastructure manager in the U.K., Network Rail [3], reported nearly 40,000 bridge strikes between 2000 and 2021. Because of their unpredictable nature, many bridge impacts go unnoticed or unreported, but the consequences can be catastrophic, resulting in structural failures or hidden damage that accelerate the bridge’s long-term degradation. In addition, extensive examinations should be conducted after every incident, causing significant delays to both road and rail users as well as disruption to the affected community.

Interactions with railroad companies in North America have uncovered a strong unmet need for a cost-effective means in response to sudden impact events, especially for two main purposes:

(1) *Early warning of impact events*: many bridge impacts go unnoticed or unreported, especially those resulting in minor or hidden damage, and the drivers or helmsmen carry on driving. More frequently, the report of impact events comes hours after the occurrence, allowing the hazard status to propagate and putting other bridge users in danger. A notable example was a collision between a river barge and a railroad bridge in Mobile, Alabama, resulting in the collapse of the bridge 20 min later as an Amtrak train crossed; the resulting accident caused 47 deaths [4]. The early warning of impact events is thus essential, which could have prevented the deaths of these individuals.
(2) *Rapid bridge condition assessment*: though many impact events are not sufficiently severe to meaningfully affect the bridge condition, once reported, mandatory post-impact inspections must be performed, according to state DOT regulations. Depending on bridge and traffic conditions, the inspection process may take from hours to days to complete, whilst bridge users are fully or partially restricted, leading to service interruptions. For example, Transportation Technology Center, Inc. conducted a study [5], finding that half of the railroad service interruptions reported were caused by collisions with bridges. Therefore, rapid bridge condition assessment is highly desired to either reduce the extended inspection time and downtime costs or allow the bridge engineers to prioritize maintenance resource allocation if the impacts are trivial.

To address the above challenges, several studies have been conducted to develop various monitoring systems for bridge impact inspection and management. Most efforts adopt wired monitoring systems and develop algorithms to detect and quantify the impact locations as well as the level of damages. Song et al. [6] developed an over-height collision detection and evaluation system for concrete bridge girders, in which piezoelectric transducers are embedded in the concrete. These sensors will trigger the network camera to take photos of collision vehicles and capture the impact energy which is used to define a damage index. With continuous power supply and data collection, wired monitoring systems are able to realize real-time impact monitoring and rapid damage assessment. Most studies are then focused on algorithm development and/or applications. Typical examples include global (multi-sensor)/local (single-sensor) identification methods for Vincent Thomas Bridge collision [7], optimal sensor placement method targeting post-collision damage detection for Ting Kau bridge [8], ship collision detection and condition assessment using visual inspection and modal analysis for Jiangyin Bridge [9], and computer vision-based ship tracking and early warning of a ship collision accident [10]. The wired monitoring system is generally expensive and hence difficult to cover beyond major bridges. In contrast, the wireless counterpart has significant potential for the pervasive monitoring of most bridges at risk. However, very limited efforts are conducted in this direction. Yang [11] developed an integrated monitoring system for impact/collision detection, including several types of sensors (e.g., strain transducers) and an inverse algorithm for damage level and impact location quantification. Wireless transmission is proposed for data collection from bridges in cold remote areas to local users; but it does not strictly belong to wireless monitoring systems, as sensors are still powered by cables. An enhanced structural health monitoring system using stream processing and artificial neural network techniques (SPANNeT) is developed by Khemapech et al. [12] for wireless sensor networks to enable real-time data streaming and proper damage detection under impacts, which, however, does not address the constraints of battery power limits for long-term deployment. Zheng et al. [13] proposed a flexible quantum tunneling composite with cushioning capability for ship-bridge collision monitoring, and simple rule-based conditions are used for damage warning. A wireless transmission system is planned for future study but has not been reported so far.

To address the limitations of the state-of-the-art solutions, this paper presents the development of *xImpact*, a cost-effective wireless impact monitoring system for civil infrastructure. It employs event-triggered wireless sensor prototypes in the authors’ previous research but extends it with the Internet of Things functionalities, such that the monitoring prototype can instrument on bridges in remote areas and automate the process of event detection and warning notification efficiently before the hazard situation propagates. Moreover, an AI-based framework is proposed to estimate the impact force location and severity, and then provide a rapid condition assessment of bridges. This feature is critical, as many impact events are not sufficiently severe to meaningfully affect the bridge condition, but once reported, mandatory post-impact inspections must be performed, placing significant demands on bridge inspectors. Therefore, rapid condition assessment is another essential feature of the *xImpact* system, to allow the bridge engineers to prioritize resource allocation for the timely inspection of the more severe impacts. Field tests were carried out to demonstrate that the developed system can capture high-fidelity synchronized structural response data under sudden impacts and enable rapid bridge condition assessment.

## 2. Monitoring System Design

There are two main challenges for data collection of bridge impact events using wireless smart sensors. The first one is the power constraint. Particularly, the wireless smart sensors must be always-on to capture unpredictable impact events, but their main power source, batteries, will be depleted quickly. The second challenge is remote data retrieval. In particular, many bridges are located far away from each other, especially in the suburbs, and conventional data retrieval requires engineers to physically travel to the base station, making it very inconvenient. A brief overview of system design to address these challenges is presented here, including on-demand sensing prototypes and cloud-based remote data retrieval development.

### 2.1. Wireless Sensor Prototypes for On-Demand Sensing

**Figure 2:**
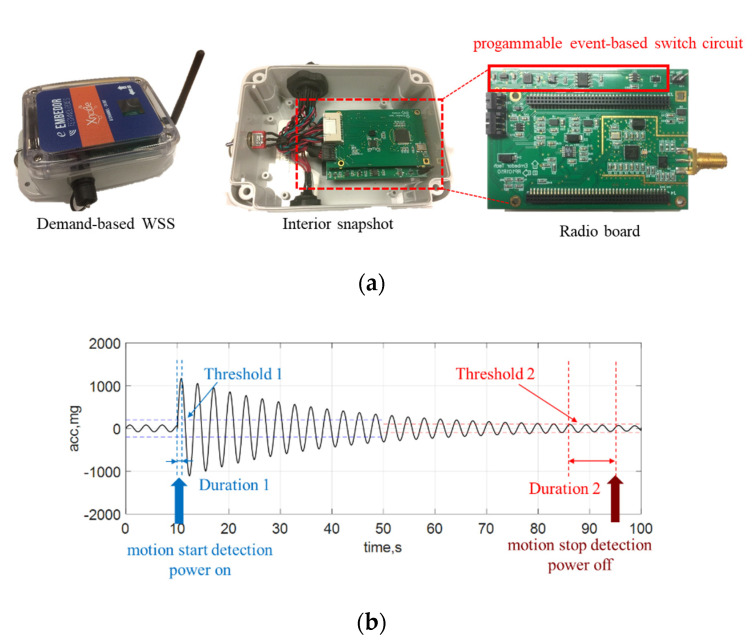
*Demand-based WSS*: (**a**) sensor prototypes, (**b**) illustration of event-triggered sensing.

This section provides a brief overview of sensing prototypes to address the challenge of power constraints for the monitoring of unpredictable impact events in bridges. To address this challenge, the *Demand-based Wireless Smart Sensors* developed by the authors [14,15,16] are adopted here for bridge impact monitoring to capture transient bridge responses. As illustrated in Figure 2a, this prototype is built upon a wireless sensor platform, Xnode [17,18], which provides high-fidelity sensing functionality via schedule-based sensing. The key novel component in this prototype to enable on-demand sensing (a.k.a., event-triggered sensing) is the programmable event-based switch circuit. Particularly, this circuit has an ultralow-power trigger accelerometer (ADXL362), a real-time clock (DS3231M), an AND gate, a latch circuit, and a MOSFET. The ADXL362 is always on to continuously monitor structural vibration. If a user-specified threshold is exceeded over a certain period, a triggering signal from ADXL362 will flip the state of the MOSFET to turn on the Xnode, as illustrated in Figure 2b. On the other hand, if the vibration is below another user-specified threshold over some time, another triggering signal will notify the Xnode to interrupt sensing and eventually power off. In sum, this circuit functions as a virtual switch to autonomously start/stop sensing without missing events of interest.

**Figure 3:**
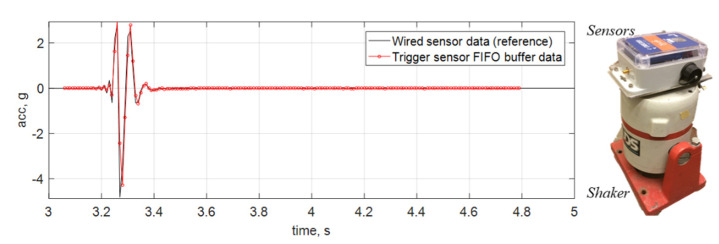
Illustration of sub-second impact detection in lab tests.

Most event-triggered sensing solutions still miss the first second of data due to cold booting of the system to full-bore data acquisition. This challenge is more critical for impact events, as they are generally short, and most significant data are possibly lost in the first second. To avoid this data loss, this prototype adopts post-sensing data fusion, where low-fidelity ADXL362 data are buffered and combined with the high-fidelity Xnode data to obtain a complete acceleration record. In this process, the ADXL362 data are first upsampled and then synchronized with the Xnode data by maximizing their cross-correlation value. More importantly, the Xnode data are used to calibrate the sampling rate of the ADXL362 data to improve their quality. More details can be found in the paper [14]. A lab test is conducted to validate the performance of this sensing prototype for short-duration impact events. In this test, a sensing prototype is attached to the top of a shaker which is used to generate a pulse signal to emulate a sub-second impact event. The prototype is turned off in the beginning; it is then automatically turned on for event monitoring when the shaker moves. The ADXL362 data are buffered surrounding the onset of this event. As can be seen in Figure 3, the prototype can successfully capture very short-duration signals without missing any data.

For the event-triggered sensing period, the power demand includes 0.3 mA for deep sleep, 170 mA for sensing, 176 mA for data processing, and 280 mA for data transmission [14]. The total power budget depends on the duration of impact events, which are usually very short, i.e., taking several seconds. For long-term deployments, considering that impact events are rare, with the proposed technology, the power demand will be averaged down a lot with that for deep sleep mode. Each sensor node is equipped with a 3.7 V DC, 10,000 mAh, rechargeable lithium battery, showing the capability of long-term deployment for about three years. In addition, each node has one solar panel to recharge its battery, significantly extending its lifetime. More discussion about power consumption can be found in [18].

### 2.2. Cloud-Based Data Management for Remote Data Retrieval

**Figure 4:**
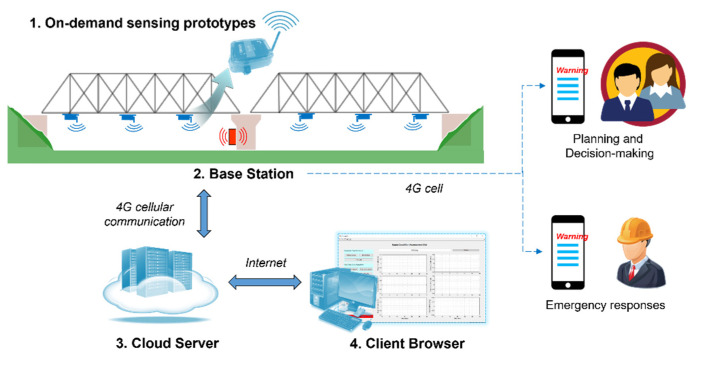
Data collection and transmission framework for bridge impact monitoring.

**Figure 5:**
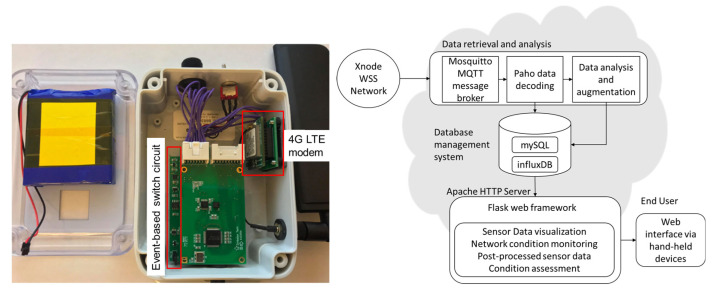
Cloud-based data management: hardware setup and framework overview [19].

This section provides a brief overview of cloud-based data collection to address the challenge of remote data retrieval for the monitoring of unpredictable impact events in bridges in large areas. To address this challenge, remote data retrieval using 4G-LTE functionality and cloud-based data management developed by the authors [19] is applied to remove human-in-the-loop for data collection. As shown in Figure 4, after the data are collected from the on-demand sensing prototypes to the base station, the base station first sends text messages to engineers for an early warning of impact events. In the meantime, it uploads data to the cloud server using a 4G LTE modem. The users can then sit in the office and download data from the cloud server through the Internet without physically visiting the bridges. To realize it, the base station is equipped with a 4G LTE modem (Sierra Wireless HL7588). This modem is mounted on an external adapter board which provides power, communication, and control pins, as shown in Figure 5. More details on hardware design can be found in the paper [19].

The cloud system is deployed on a Virtual Private Server (VPS) with 2 vCPUs, 4 GB of RAM, and 80 GB of hard disk to host this cloud system. As illustrated in Figure 5, this server actively waits for data from the sensor network through a Message Queuing Telemetry Transport (MQTT) data broker. Once an event is detected on the sensor network, the data are sent to this broker. Then another MQTT client processes and decodes the raw data and then stores them into respective databases for further analysis. Finally, the processed data are presented and ready to be queried at the front end of the web interface. The MQTT protocol follows a publish/subscribe (pub/sub) scheme. This scheme contains clients that can publish (send) and subscribe (receive) to messages belonging to one or multiple topics. The broker receives the messages from the publisher and distributes them to the subscribers according to their subscribed topics [20]. In this application using the MQTT protocol, the sensor network is a publisher, and the cloud server is a data broker and a publisher. The data publisher implemented on the base station sends sensor data (Monitoring Data topic) and the network status containing the whole network current and voltage information (Status Data topic). On the cloud server, the broker receives the data from the base station and distributes it to the data processing and storage pipeline. To ensure proper performance for managing various types of data collected from the monitoring system, MySQL is implemented to handle relational data, and InfluxDB, an open-source schemaless database, is chosen for time-series database management. Both database systems are developed to work as MQTT subscribers through a Python data parser. Any dataset obtained from the sensor network is distributed to the parser, which then separates into several groups, including Sensor Information, Sensor Data, and Status Data.

## 3. Rapid Condition Assessment

While the proposed system has the essential functionalities to detect bridge impact events and capture structural responses, a missing brick is a strategy for the impact localization and damage severity estimation using measurement data, which, however, is difficult. Lu et al. [21] found that bridge damage under impact events can be classified into two categories: local damage and global damage. Local damage, e.g., cracks, concrete crush, and reinforcement yielding, is positively correlated to peak impact force. Global damage, e.g., distortion, bending failure, and girder falling, is strongly related to impulse. Accordingly, both peak impact force and impulse can be used as two crucial indicators to infer bridge damage under impact events. This section presents a rapid condition assessment framework for bridge impact monitoring.

### 3.1. Framework Overview

**Figure 6:**
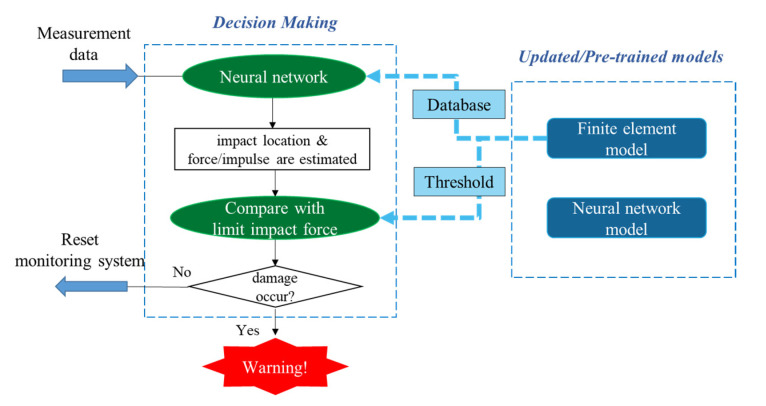
AI-based rapid condition assessment framework.

The detailed flowchart of rapid condition assessment is illustrated in Figure 6. After being deployed for bridge impact monitoring, the wireless sensor nodes are often turned off to save the limited battery power. When an impact event occurs, the sensor nodes are quickly turned on to start sensing immediately. Once the event ends, the sensor nodes stop measurement and perform post-event time synchronization. Afterward, the obtained bridge acceleration responses are transmitted back to the base station. In the base station, the measurement data goes through a neural network model to estimate the impact information, including the impact force (peak force and impulse) and the impact location. Finally, the obtained impact force is compared with the maximum limiting impact force that will not introduce damages at the corresponding location. Note that the limiting impact force is obtained from the numerical analysis, and it is determined to be the minimum impact force that causes plastic deformation in the nonlinear analysis. If the estimated impact force is larger than the limiting impact force, the bridge is suspected to be damaged. Then, warning messages will be sent to the engineers to propose informed decisions in an efficient manner. Otherwise, the system resets itself, and all the nodes are turned off to save energy and wait for the next event. To achieve the proposed strategies, two models need to be established before testing, including a finite element model (FEM) and an artificial neural network (ANN), which will be discussed in the following sections. Note that the system is designed to detect moderate or significant events with reasonably large magnitude, and it may not be able to detect two events happening closely to each other.

### 3.2. Finite Element Model Development

To realize the rapid condition assessment of bridge impact monitoring, a reliable finite element model must be built and updated to represent the real bridge in terms of dynamic responses. It serves two purposes: (1) to build the learning database for neural network modeling, and (2) to obtain maximum allowable impact forces at different bridge locations through nonlinear analysis.

In particular, an initial FE model is first built based on the drawings in a commercial software (e.g., ABAQUS). Taking a suspension bridge as an example, the model consists of several components: (1) suspension cables are modeled using truss elements; (2) bridge beams and columns are modeled using beam elements; and (3) decks are modeled using solid elements. To consider material nonlinearity, a bilinear stress-strain curve is employed for steel components. It should then be updated using field test data to match the dynamic properties with the real bridge. In particular, a full-scale dynamic test should be performed to obtain the modal properties (i.e., natural frequency and mode shapes). Model updating is then applied to optimize the dimensions and other parameters of the FE model, such that the difference in modal properties between the field test data and the FE model data are minimized. The process of FE modelling and updating follows the methodology described in the paper [22]. In particular, the objective function for model updating is defined as,

(1) $$f\left(p\right)=\eta \overset{n_{f}}{\underset{i=1}{\sum }}{\left[\frac{\varpi_{id,i}-\varpi_{fe,i}\left(p\right)}{\varpi_{id,i}}\right]}^{2}+\beta \overset{n_{m}}{\underset{i=1}{\sum }}\frac{\theta_{i}}{\pi /2}$$

where ϖ*_id,i_* is *i*th identified natural frequencies from the measured data; ϖ*_fe,i_* is *i*th natural frequency from the FE model; *η* and *β* are weighting constants that are assumed to be unity in this study; *n_f_* and *n_m_* are the number of natural frequency residuals and number of mode shape residuals, respectively; and $\theta_{i}$ is defined as $cos^{-1}\left(\sqrt{{\mathrm{MAC}}_{i}}\right)$, where MAC is defined as [23]

(2) $${\mathrm{MAC}}_{i}=\frac{{\left(\Phi_{fe,i}^{T}\Phi_{id,i}^{T}\right)}^{2}}{{\left(\Phi_{fe,i}^{T}\Phi_{fe,i}^{T}\right)}^{2}{\left(\Phi_{id,i}^{T}\Phi_{id,i}^{T}\right)}^{2}}$$

where Φ*_id,i_* is *i*th identified mode shape from the measured data; Φ*_fe,i_* is *i*th mode shape from the FE model.

**Figure 7:**
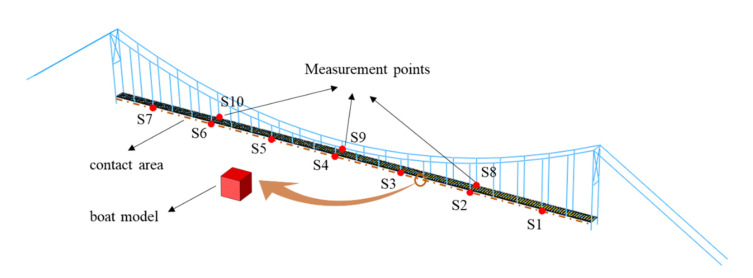
Bridge/ship impact analysis illustration.

The updated model is employed for the simulation of a bridge/ship impact, in which a boat is traveling at a certain speed and hitting the bridge horizontally. This is a highly nonlinear dynamic process. The performance of a boat is not the focus in this study. So, it can be simplified as a cube using solid elements. The contact between the bridge and the boat is defined using standard surface-to-surface contact. The contact area in the bridge model is defined in the longitudinal girders horizontally. Various initial boat speeds give rise to different values of peak impact force. Different impact locations are simulated along the bridge, starting from the mid-span to the end. The initial boat location is adjusted in each analysis, such that the boat always hits the bridge at the same time for the sake of data collection. Three-axis accelerations are recorded at selected points on the bridge in the analysis. The impact force is estimated by multiplying the stress value in the boat and cross-section area of the boat, and the impact impulse is obtained by summing the area under the curve of the impact force against time. The illustration of impact analysis can be seen in Figure 7.

In this study, model updating should be conducted if significant differences exist between the FE model and the real bridge using dynamic field test data. First of all, when a model is built initially according to drawings, it should be updated to match with the real structure. In addition, when several damages are identified or repair is further conducted after an impact event, another round of model updating should be considered after dynamic field tests. In addition, loading conditions may also need be considered for model updating of railroad bridges, because the mass of a train is comparable with that of the bridges. However, it may not be very critical, as the probability for the case in which both an impact and a train crossing take place on the same railroad bridge is very low.

### 3.3. Neural Network Model Development

An ANN model is established to process impact measurement data and identify impact location and impact force information, which can be further used for the rapid condition assessment of bridges under impact events. Following the impact signal processing strategies developed for aircraft structures [24,25], important features are extracted from the bridge response measurements as inputs for the ANN model.

Specifically, features for impact location detection include: (1) time of arrival of vibration signals (ToA), (2) maximum acceleration record, (3) time at maximum acceleration record, (4) maximum envelope of acceleration record, and (5) time at the maximum envelope of acceleration record. These features are considered to contain adequate information on impact location. For example, after an impact occurs, similar to wave propagation, the induced vibration evolves earlier at the measurement point that is closer to the impact location. Accordingly, the arrival time of vibration signals is employed for impact location detection, and it is defined when the absolute acceleration first exceeds a user-defined threshold (e.g., 40 mg is chosen for impact detection in this study). Specifically, ToA at measurement point *i* is expressed as,

(3) $${\mathrm{ToA}}_{i}=t_{i}-\min(t)$$

which is the offset between the arrival time at point *i* and the smallest arrival time within all the points. In addition, the impact energy distributes unevenly, indicating that the maximum absolute acceleration record is larger if the measurement point is closer to the impact location. Therefore, both the maximum acceleration record and maximum envelope of the acceleration record are calculated for impact location detection. To obtain the signal envelope, a Hilbert transform is performed for each acceleration record *a(t)*,

(4) $$H\left(a\left(t\right)\right)=\frac{1}{\pi }\int_{-\infty }^{\infty }\frac{a\left(\tau \right)}{t-\tau }d\tau$$

The real part of the transformed result is the original real data, whilst the imaginary part is the actual Hilbert transform. The magnitude of the transformed result is the envelope of the acceleration record. Though the acceleration record contains three-axis measurement data, only data in the vertical axis and lateral axis is processed for impact events to generate features, respectively; the data in the longitudinal axis has a very small amplitude, and hence it is neglected. Therefore, a total of 10*N* features obtained from *N* measurement points at two axes are used as inputs for the ANN to detect impact locations.

Features for impact force identification include: (1) maximum acceleration record, (2) time at maximum acceleration record, (3) the maximum value of detailed coefficients after discrete wavelet transform (WT) using level 4 Daubechies wavelet (db4), (4) the maximum value of approximated coefficients of WT (db4), as well as (5) detected impact location. The first four types of features have been considered effective for impact force reconstruction in the paper [25]. They are extracted from *N* measurement points at the vertical axis and lateral axis, respectively. The last feature is the identified impact location from the ANN, which can help to increase the accuracy of impact force estimation. In sum, a total of 8*N* + 1 features is extracted for impact force estimation, which includes peak impact force and impact impulse. The summary of inputs for impact information identification is listed in Table 1.

**Figure 8:**
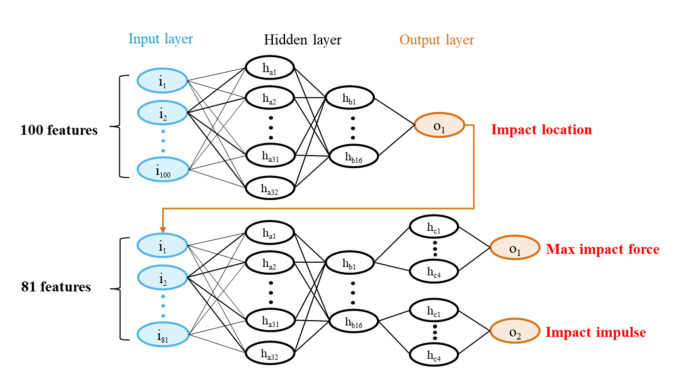
Artificial neural network architecture for impact force estimation.

The neural network model is established using multi-layer perception in Keras using Python. The ANN model contains two subnetworks: (1) one for impact location detection, and (2) one for impact force estimation. In particular, the first subnetwork has one input layer, several fully connected hidden layers, and one output layer. The result from the output layer of the first subnetwork is subsequently used as the input for the second subnetwork. The second subnetwork has a similar architecture to the first one, but the last hidden layer of the second subnetwork is split into two parts, one for max impact force estimation and one for impact impulse estimation, respectively. Between adjacent layers, the neuron behaviors were defined and tested using different activation functions (e.g., ReLU and sigmoid). The loss function is defined using mean squared error, and the metric function is defined using mean absolute error to judge the performance of the trained ANN model. The two subnetworks are trained independently. Because the second subnetwork has two different outputs which have different scales, the weight value of the loss function is defined for each output as α and β.

(5) $$Loss\;function=\alpha \cdot MSE_{pf}+\beta \cdot MSE_{m}$$

where *MSE_pf_* is the mean squared error of peak impact force; *MSE_m_* is the mean squared error of impact impulse. The relative value of weight value can be adjusted based on the ratio of $MSE_{pf}$ and $MSE_{m}$. To identify an optimal ANN, different topologies of the ANN are trained and tested, containing various numbers of hidden layers as well as various hidden neurons per layer. The finalized network architecture is determined to achieve the best performance of testing, as illustrated in Figure 8. Particularly, the first subnetwork has two hidden layers, one with 32 neurons and the other with 16 neurons; the second subnetwork has three hidden layers, the first two layers have the same architecture as the first subnetwork, whilst the last layer is divided into two 4-neuron parts. In addition, the RELU is employed as the activation function for all the layers.

## 4. Full-Scale Demonstration

### 4.1. Testbed Description

**Figure 9:**
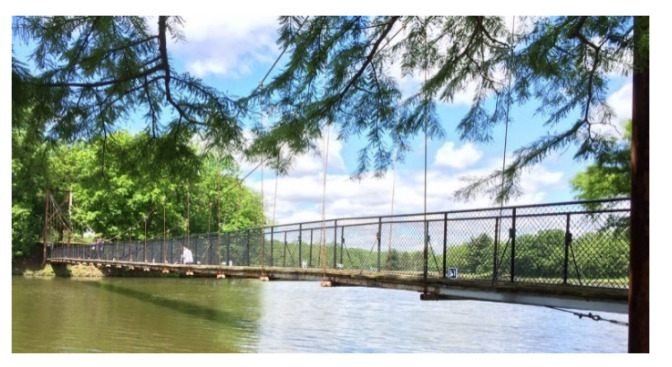
Suspension bridge in Lake of the Woods, Mahomet, IL, USA.

A single-span pedestrian suspension bridge located over Lake of the Woods in Mahomet, Illinois, is considered the testbed for bridge impact monitoring, as shown in Figure 9. The clear span of the bridge is 67 m, with two bridge towers standing at each side of the lakeshores. The bridge contains two longitudinal girders and two suspended cables. The timber deck is supported on a series of 21 steel beams hanged by 42 suspenders. Boating is very popular in the Lake of the Woods, especially near the bridge where the boat peninsula is located. Therefore, one of the main threats of concern for the aging bridge is the collision between boats and the bridge. Installing a monitoring system is of great help to provide early warning of the bridge/boat collision and conducting rapid condition assessment. Accordingly, managers from the County Forest Preserve District can conduct emergency responses and informed maintenance decisions (e.g., bridge closure for safety concerns or sending maintenance technicians).

### 4.2. Finite Element Modeling and Updating

**Figure 10:**
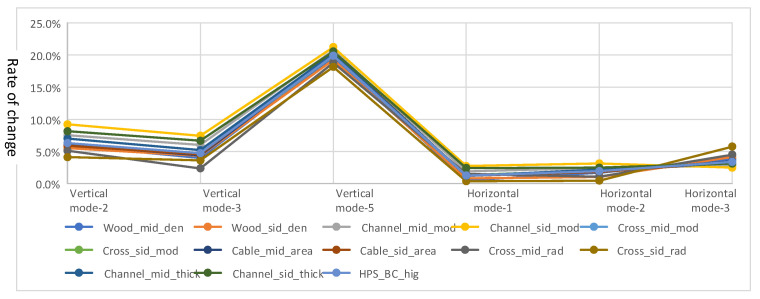
Sensitivity of main parameters measured by six natural frequencies. Note: The density (den), Young’s modulus (mod), area of section (area), thickness of the steel plate (thick), radius of cables (rad), and height of the section (hig) of different parts of the bridge model were tested. The structure from middle 1/3 span is named as ‘mid’; The rest parts are named as ‘sid’; The beams between towers are named as ‘HPS_BC’. Wood is the bridge deck; Channel is the side steel beam of the bridge; Cross is the X brace between side beams; Cable is the main cable of the suspension bridge.

A bridge FE model created by Hoskere et al. [26] in ABAQUS is employed as the initial FE model. Severe corrosion was found in many components of the real bridge, such as main girders, braces, gusset plates for connections, beams, and columns in the bridge tower. Accordingly, model updating must be performed to match the dynamic behavior between the FE model and the real bridge. The main strategy of model updating is to adjust Young’s modulus, the geometry of the bridge components, as well as the density of wood decks, which changes when it becomes wet. Before model updating, all the parameters are examined in the sensitivity analysis to select the most appropriate ones. As shown in Figure 10, when the parameters decreased to 90% of the initial level, the sensitivity of the main parameters is measured by the change of six main natural frequencies. Note that these six natural frequencies are observable during field tests, including mode 2, 3 and 5 in vertical direction and mode 1, 2 and 3 in horizontal direction, as shown in Figure 10. The results show that Young’s modulus of middle channels, radius of middle cross-section and side cross-section, and thickness of side channel steel plate affect the modal properties most and are used in following model updating process.

**Figure 11:**
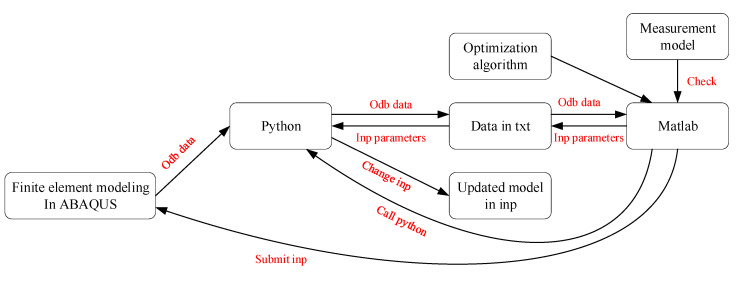
Model update framework with ABAQUS, Python and MATLAB.

**Figure 12:**
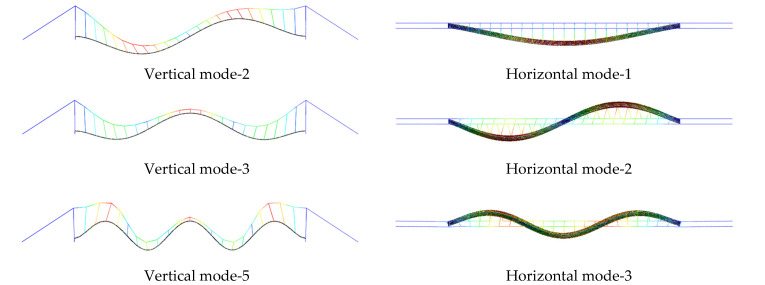
Updated FE model dynamic mode shapes.

To implement model updating, ABAQUS, Python and MATLAB are linked together in the process to leverage their benefits and tools, as shown in Figure 11. First, we establish the initial finite element model and generate the initial Inp file in ABAQUS. When a finite element calculation is completed, the Odb file is post-processed in Python to extract the structural vibration parameter. The command ‘fmincon’ in the Optimization Toolbox of MATLAB was adopted to update the model parameters by comparing the vibration characteristics of the FE model with the measured vibration characteristics. Then, the Inp file was updated to complete one round of model updating by adjusting the selected parameters. This process is repeated until the natural frequency error, and MAC error are minimized. The dynamic properties from the updated model and the measured data are compared in Table 2 and Figure 12. Their differences are considerable, especially for horizontal modes, which are dominant modes during bridge/ship impacts.

### 4.3. Impact Simulation and Impact Force Safety Limitation

Besides building a database for ANN training and testing, the impact simulation is also employed to obtain the maximum allowable values of impact force/impulse. In particular, a series of nonlinear impact analysis is performed at various locations of the bridge. The positions of impact analysis include all the brace joints (collocated with suspenders) and the intermediate midpoints between joints (located on the girders). The force that gives rise to the maximum bridge stress of equal to steel yielding stress is considered as the maximum allowable impact force. The corresponding impulse is considered as the maximum allowable impulse.

**Figure 13:**
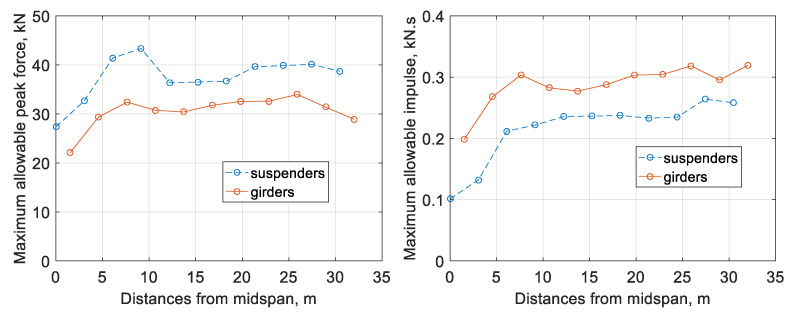
Maximum allowable impact force and impulse.

Assuming that the bridge is symmetric about its midspan, only the right half-span of the bridge is investigated. The maximum allowable impact force/impulse varies at different locations of the bridge are collected accordingly. The summary of these values is presented in Figure 13. The impact locations are divided into two categories: suspenders and main girders, because they have different local stiffness and hence different responses. In particular, suspenders have larger local stiffness than girders. If the impact is collocated with the suspenders, the peak impact force is larger than adjacent girders; however, the contact time between the boat and the bridge is shorter, which results in a smaller impulse than adjacent girders.

### 4.4. Neural Network Model Training and Testing

**Figure 14:**
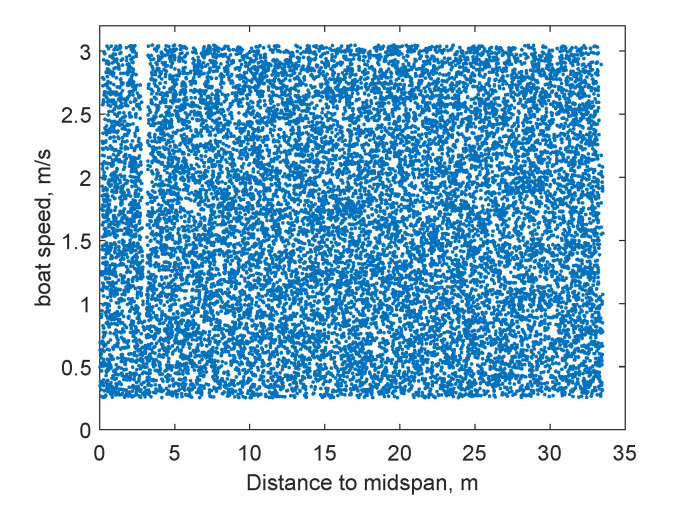
Learning database for ANN training and testing.

In addition to recording the maximum allowable impact force, three-axis accelerations are collected at a total of 10 points on the bridge in the impact analysis, as illustrated in Figure 7. They are used to build a database for ANN training and testing. In particular, the boat model hits the bridge horizontally at a random location and at a random speed, which is only conducted within the right half-span of the bridge, by taking advantage of bridge symmetry. The initial boat speed is set to be a random value between 0.25 m/s and 3.00 m/s. Boat impacts with varying initial speeds result in different impact forces at different locations. The specific speed range is intentionally configured to include both linear (undamaged) and nonlinear (damaged) scenarios in the database. Three-axis acceleration recording is collected at 100 Hz for each measurement point, with a total length of 6 s. During impacts, the force history is also recorded to calculate the peak impact force and impact impulse. The initial location of the boat model is adjusted to ensure that the bridge/boat collision starts at 0.01 s. A total of 16,895 data sets with varying impact locations and initial boat speeds are obtained through a series of numerical simulations, serving as the learning database for ANN training and testing, detailed in Figure 14. The data sets are randomly split into 75% for training and 25% for testing.

**Figure 15:**
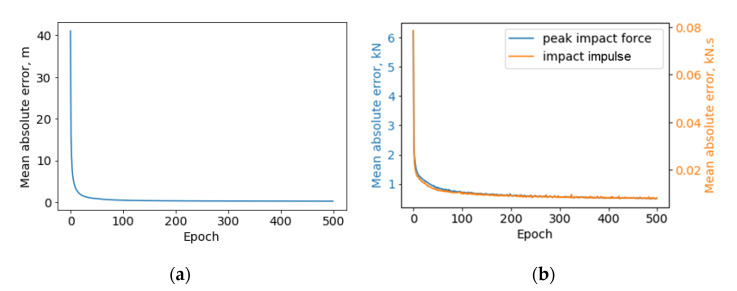
Artificial neural network learning curves: (**a**) impact location, (**b**) impact force.

Figure 15 presents the learning curves of two subnetworks in the finalized ANN model, both of which converge to a very low rate, demonstrating that the number of epochs is adequate to achieve high accuracy. The accuracy of the trained ANN is expressed through mean absolute error and summarized in Table 3. Specifically, the average of absolute value for test errors is shown in the table. To provide a rough idea of how big the test absolute errors are, the mean value and standard division of the overall learning database (containing 16,895 data sets) are also provided in the table. The error is satisfactory for rapid condition assessment of bridges.

### 4.5. Field Test and Results

To validate the capabilities of the developed system and the efficiency of the proposed rapid condition assessment framework, a full-scale demonstration is performed on the pedestrian bridge in Lake of the Woods in Mahomet, Illinois. The impact is generated by a large-sledge impulse hammer in the horizontal direction. The hammer, model PCB086D50 (PCB Piezotronics, Inc., Depew, NY, USA), is equipped with a force sensor on the tip.

**Figure 16:**
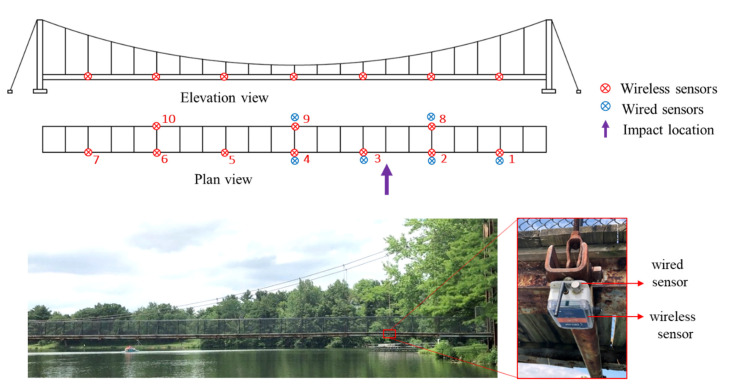
Sensor node deployment for bridge impact monitoring.

The monitoring system is deployed on the bridge, as shown in Figure 16. A total of 10 *Demand-based WSSs* were installed on the beam joints beneath the bridge. They were attached to the steel beams using magnets. Another wireless sensor is deployed far away from the bridge, serving as the base station. The threshold for event-start detection was configured to be 40 mg over 0.02 s; the threshold for event-stop detection was set to be 10 mg over 5 s. In addition, the sampling rate for high-fidelity measurement was 100 Hz. For comparison, six uniaxial wired accelerometers, model PCB353B33, were selected as reference sensors and collocated with wireless sensors on the right half-span bridge. They were mounted horizontally on the enclosure of wireless sensors using hot glue. Both the wired and force sensors on the impact hammer were connected to a DAQ system, VibPilot, (m + p international). The sampling rate was 8192 Hz, aimed to capture the transient peak of force sensor signal. The measurement data from wired sensors was later decimated to 100 Hz for a fair comparison with the wireless sensor data. In addition, the measurement mode of the DAQ system was a pulse-triggered mechanism, i.e., the system started measurement when it detected a pulse like signal in the force sensor.

**Figure 17:**
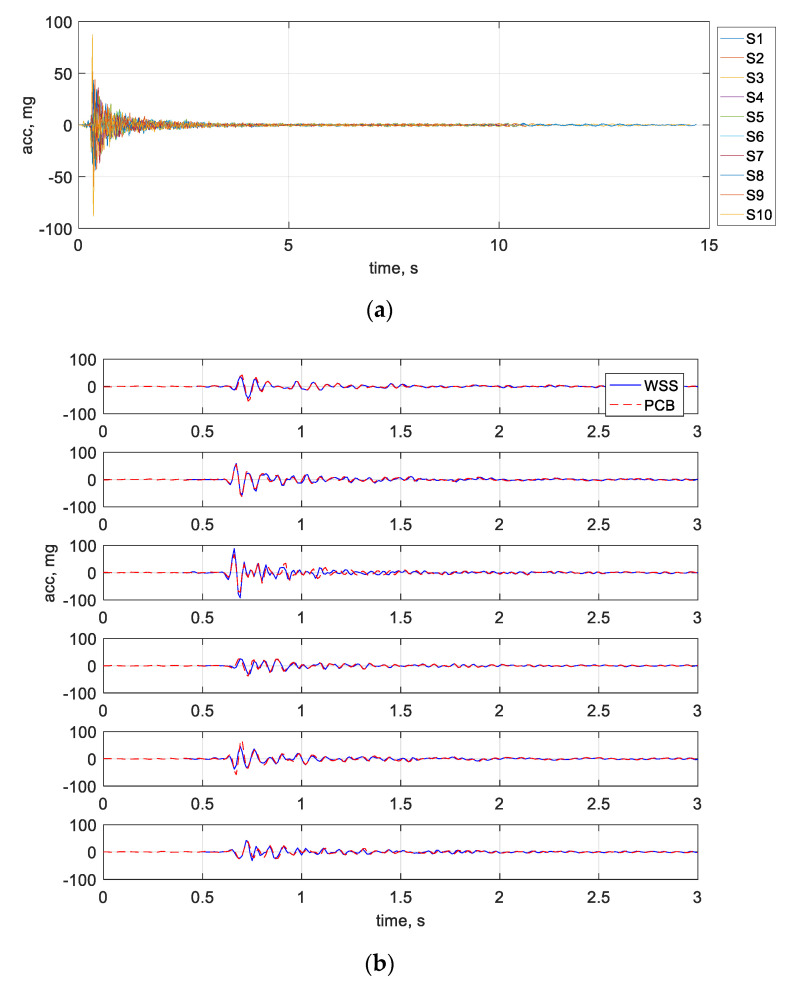
Impact response measurements: (**a**) vibration responses from WSS. (**b**) measurement comparison for node 1, 2, 3, 4, 8, 9.

After the impact occurred, the *Demand-based WSS* woke up and started the measurement. In the meantime, the DAQ system was triggered to collect data from the wired sensors and the impact hammer. Figure 17 shows the comparison between the bridge responses from the developed system and the wired sensors. As can be seen, the most critical data has a short duration of fewer than 3 s. The developed monitoring system successfully captured the onset of impact events and obtained complete bridge responses. The measurement data match very well with the wired sensor data, demonstrating that the developed system provided high-quality synchronized data.

The obtained measurement data are preprocessed, and the extracted features are then fed into the pretrained ANN model. The impact information is identified and summarized in Table 4 (labeled as Test 1). The error is slightly larger than the testing error in Table 3, but they are satisfactory and in a reasonable range. Specifically, the distance between two suspenders is 3 m, and if the error of impact location detection is 2 m, it is still helpful for bridge owners to identify the location between two suspenders. In addition, the impact force is much smaller than the maximum allowable impact forces, indicating that there is no need to send warning messages to upset bridge owners if an impact event occurs. To demonstrate the utility of the system, one additional test is conducted at different impact locations, and the estimation is also included in Table 4 (labeled as Test 2).

It should be clarified that, in the tests, the impact from the hammer is set intentionally to be low to prevent any potential damage to the pedestrian bridge, and hence the waking up threshold of each sensor is low. In long-term deployment, the configuration of each node should be adjusted to minimize undesired waking up for events of no interest, e.g., people jumping.

To further evaluate the proposed system, a comparison is drawn between the proposed system and other existing systems. As listed in Table 5, the existing wireless monitoring systems are less capable than their wired counterparts, mainly due to the challenges identified in Section 2. The proposed system is the only cost-effective solution that is capable to meet the needs of early warning and rapid condition assessment of bridge impacts.

## 5. Conclusions

In this paper, the unmet needs of cost-effective monitoring systems for bridge impacts have been discussed, including the early warning of impact events and rapid bridge condition assessment. To address these needs, an intelligent wireless monitoring system, *xImpact*, is developed as a cost-effective solution. In this system, on-demand sensing prototypes and cloud-based data retrieval are adopted to address the challenges of power constraints and remote data retrieval for bridge impact monitoring, hence realizing the objective of early warning. Furthermore, the proposed system includes an AI-based rapid condition assessment framework to estimate impact location and impact force, and further evaluate damage severities after impacts. Finally, the proposed system is demonstrated through a full-scale field test. It can be claimed that the system can detect bridge impacts, capture high-fidelity synchronized data, retrieve data remotely, and support rapid condition assessment of bridges. The test results show that it can achieve impact localization with the error of around 2 m, and impact force estimation with the error of 1 kN for peak force and 0.01 kN·s for impulse. Though this study has demonstrated the capability of the proposed system, more additional tests and long-term deployment should be carried out in the future to evaluate the reliability of the system.

## Figures and Tables

**Table 1:** Features extracted for impact force estimation.

No.	Feature	Usage	
		Location Detection	Force Estimation
1	time of arrival of vibration signals	√	
2	maximum acceleration record	√	√
3	time at maximum acceleration record	√	√
4	maximum envelope of acceleration record	√	
5	time at maximum envelope of acceleration record	√	
6	maximum value of detailed coefficients (WT-db4)		√
7	maximum value of approximated coefficients (WT-db4)		√
8	identified impact location		√

**Table 2:** Model updating results to represent the real bridge.

Modes		Measurement (Hz)	Original Model (Hz)	Updated Model (Hz)	Updated Error (%)	Original MAC(%)	Updated MAC (%)
Vertical modes	2	0.652	0.501	0.634	2.84	80.8	99.2
	3	1.073	1.045	1.139	−5.79	80.4	97.6
	5	2.735	2.738	2.685	1.86	71.0	93.8
Horizontal modes	1	0.845	1.290	0.847	−0.24	93.3	99.1
	2	1.411	2.009	1.477	−4.47	81.6	95.7
	3	1.867	3.328	1.735	7.61	50.5	91.3

**Table 3:** Neural network model testing results.

Impact Info	Learning Database		Testing Average Error
	Mean	Std	
Location (m)	50.45	9.55	0.28
Peak force (kN)	11.17	8.56	0.54
Impulse (kN·s)	0.25	0.12	0.01

**Table 4:** Artificial neural network prediction results.

Impact Info	Test 1			Test 2		
	Test	Estimation	Error = |Test − Estimation|	Test	Estimation	Error = |Test − Estimation|
Location (m)	45.72	47.76	2.04	48.77	47.76	1.01
Peak force (kN)	3.13	4.07	0.94	3.59	4.65	1.06
Impulse (kN·s)	0.03	0.04	0.01	0.04	0.05	0.01

**Table 5:** Comparison of bridge impact monitoring systems.

Studies	Sensor Type	Event Detection	Impact Localization	Impact Force Estimation	Damage Evaluation	Lab/Field Test
Song et al. [6]	Wired, PZT sensor	Yes	No	Yes	Yes	Lab
Yun et al. [7]	Wired, acceleromter	Yes	No	No	Yes	Field
Ye et al. [10]	Wired, camera	Yes	Yes	No	No	Field
Yang [11]	Wired, strain gauge	Yes	Yes	Yes	No	Field
Khemapech et al. [12]	Wireless, strain gauge	Yes	No	No	Yes	Field
Zheng et al. [13]	Wireless, QTCs sensor	Yes	Yes	No	No	Lab
This study	Wireless, accelerometer	Yes	Yes	Yes	Yes	Field

## Data Availability

Not applicable.
